# Comparative and Phylogenomic Evidence That the Alphaproteobacterium HIMB59 Is Not a Member of the Oceanic SAR11 Clade 

**DOI:** 10.1371/journal.pone.0078858

**Published:** 2013-11-01

**Authors:** Johan Viklund, Joran Martijn, Thijs J. G. Ettema, Siv G. E. Andersson

**Affiliations:** Department of Molecular Evolution, Biomedical Centre, Science for Life Laboratory, Uppsala, Sweden; The University of Hong Kong, China

## Abstract

SAR11 is a globally abundant group of Alphaproteobacteria in the oceans that is taxonomically not well defined. It has been suggested SAR11 should be classified into the novel order Pelagibacterales. Features such as conservation of gene content and synteny have been taken as evidence that also the divergent member HIMB59 should be included in the order. However, this proposition is controversial since phylogenetic analyses have questioned the monophyly of this grouping. Here, we performed phylogenetic analyses and reinvestigated the genomic similarity of SAR11 and HIMB59. Our phylogenetic analysis confirmed that HIMB59 is not a sister group to the other SAR11 strains. By placing the comparison in the context of the evolution of the Alphaproteobacteria, we found that none of the measures of genomic similarity supports a clustering of HIMB59 and SAR11 to the exclusion of other Alphaproteobacteria. First, pairwise sequence similarity measures for the SAR11 and HIMB59 genomes were within the range observed for unrelated pairs of Alphaproteobacteria. Second, pairwise comparisons of gene contents revealed a higher similarity of SAR11 to several other alphaproteobacterial genomes than to HIMB59. Third, the SAR11 genomes are not more similar in gene order to the HIMB59 genome than what they are to several other alphaproteobacterial genomes. Finally, in contrast to earlier reports, we observed no sequence similarity between the hypervariable region HVR2 in the SAR11 genomes and the region located at the corresponding position in the HIMB59 genome. Based on these observations, we conclude that the alphaproteobacterium HIMB59 is not monophyletic with the SAR11 strains and that genome streamlining has evolved multiple times independently in Alphaproteobacteria adapted to the upper surface waters of the oceans.

## Introduction

The world’s oceans are dominated by the SAR11 clade of the Alphaproteobacteria [1], which are key players in the ocean carbon cycle and represent about 25% of cells in coastal, estuary and open-ocean habitats [2,3]. Genome sizes are in the 1.4–1.6 Mb range with an estimated core of about 500 genes [4,5]. It has been hypothesized that the downsizing of the SAR11 genomes was driven by selection for an efficient utilization of the limiting resources available in the oceans [4,6]. This process is thought to have occurred independently of the reduction of genome size in the Rickettsiales (Viklund et al. 2012) that also belong to the Alphaproteobacteria and have genomes in the 1–2 Mb range. The streamlining hypothesis for the evolution of the SAR11 clade suggests that selection has favoured efficient transport systems and a small cell volume of only 0.01 µm so as to concentrate nutrients inside the cell and thereby increase substrate-processing rates [4,6,7].

 Despite its abundance and global importance, the SAR11 group of bacteria is taxonomically not well defined. One school of thought suggests that they are affiliated with the Rickettsiales [8–10], whereas others argue that this placement is an artefact of a biased mutation pressure towards AT in the two lineages [11–13]. The alternative hypothesis is that SAR11 is most similar to environmental Alphaproteobacteria with larger genomes, as inferred from phylogenetic analyses based on conserved and less biased genes [11–13].

 It was recently suggested that the SAR11 clade should be classified as a novel order, called the “Pelagibacterales”, and that one of the subclades, which contains the type strain *Candidatus* Pelagibacter ubique, should be considered a new genus called “Candidatus Pelagibacter” [5]. However, the diversity of the 16S rRNA sequences within the proposed order Pelagibacterales is very high and includes more than a dozen ecotypes and five different subtypes [5]. Genome sequence data is available for seven isolates, five of which fall within subclade Ia. Nucleotide sequence identity within subtype Ia is high, > 98% 16S rRNA sequence identity, i.e. well above the threshold normally used for species designations. Strain HIMB114 has been classified into subtype IIIa and is considerably more divergent with a 16S rRNA sequence identity of 88% to *Ca*. Pelagibacter ubique. The most divergent member, HIMB59, has been classified into subtype V and is only 82% identical at the 16S rRNA level to *Ca*. Pelagibacter ubique. Despite the divergence in sequence similarity, genome sizes are in the range of 1.3 to 1.5 Mb for all isolates, with genomic G + C content levels spanning from 29% to 32%, with the subtype Ia genomes having the lowest G + C content and HIMB59 the highest. 

 The suggested monophyly of HIMB59 with members of subtype Ia and III of the SAR11 group of bacteria [5,9] has been questioned [13]. The controversy has arisen because phylogenetic analyses suggest that HIMB59 should be affiliated with the Rhodospirillales rather than the SAR11 clade [13]. Despite its disputed phylogenetic position, it has been argued that HIMB59 should be placed within the order Pelagibacterales because of similar gene contents, gene orders and conservation of the HRV2 region across all isolates [5]. Here, we re-examine the genomic similarities of the SAR11 clade and suggest that HIMB59 should not be considered a member of the Pelagibacterales.

## Results

### Phylogenomics

**Figure 1:**
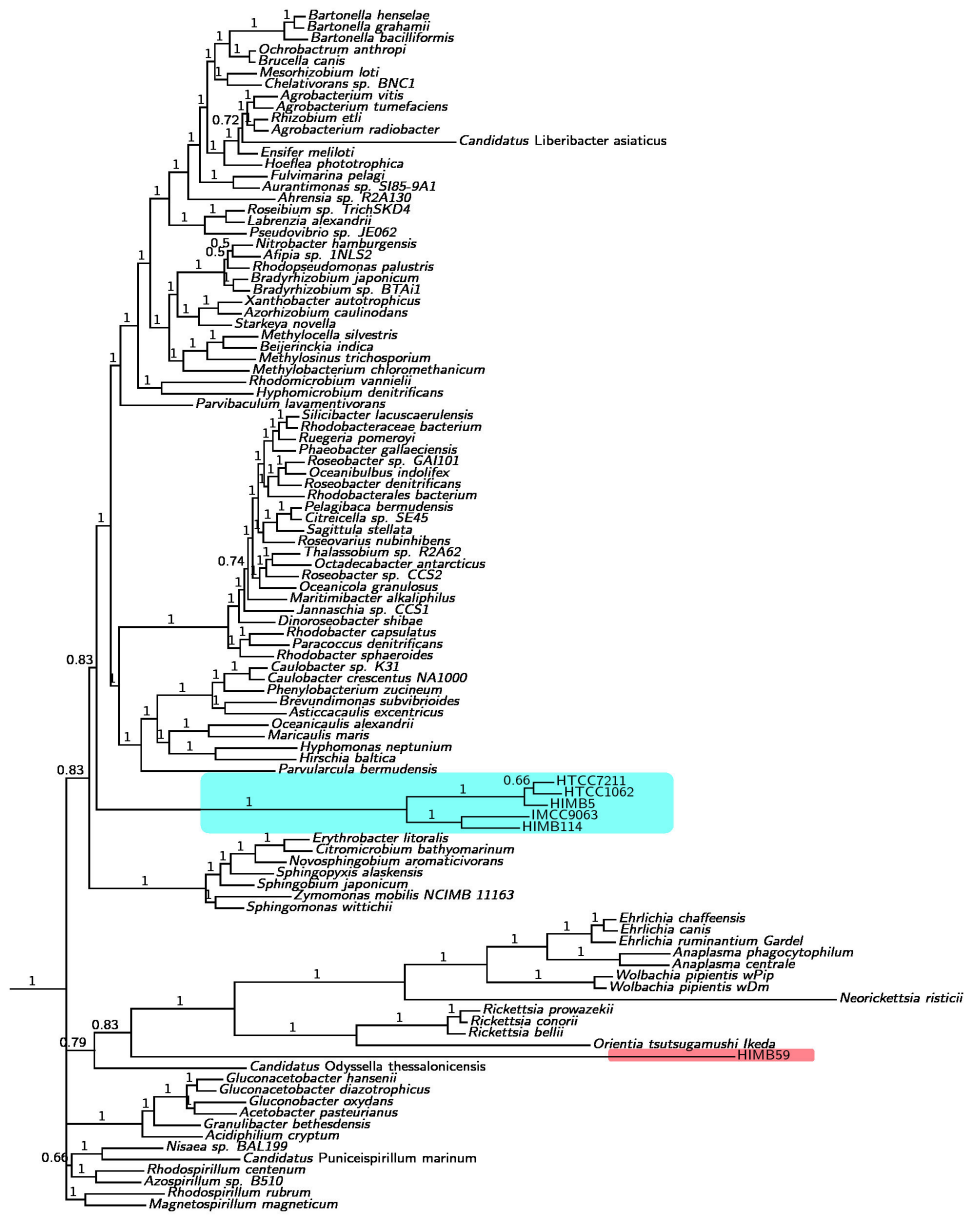
Phylogenetic analysis of the Alphaproteobacteria. Bayesian tree inferred with the CAT model applied to an alignment of 150 concatenated pan-orthologous proteins. HIMB59 (marked in red) clusters with the Rickettsiales. The SAR11 clade (marked in blue) is placed within a broad group of free-living alphaproteobacterial species that includes the Spingomonadales, Rhodobacterales, Rhizobiales, and Caulobacterales. Numbers at nodes show PP values.

**Phylogenomic analyses of Alphaproteobacteria indicate that HIMB59 is not affiliated with SAR11**. To re-examine the relationship of HIMB59 to the SAR11 genomes, we repeated our previous phylogenetic analysis of alphaproteobacterial genomes [12], and included several isolates of the SAR11 clade. As representatives of subtype Ia of the SAR11 clade we selected HTCC1062, HTCC7211 and HIMB5, and as representatives of subtype IIIa we included HIMB114 and IMCC9063 (Table 1). This resulted in a dataset of 135 alphaproteobacterial genomes that were clustered into orthologous clusters (OC) using orthoMCL (Table S1). We first constructed a phylogeny based on an alignment of a concatenated dataset of 150 proteins (Figure 1; Figure S1). As in our previous analyses [12], we noted that the placement of the SAR11 clade was sensitive to the method used to infer the phylogeny. With the Bayesian method and the CAT model the SAR11 clade clustered with the free-living Alphaproteobacteria (Figure 1), whereas it clustered with the Rickettsiales with the maximum likelihood method (Figure S1). Also the placement of HIMB59 was sensitive to the use of method such that it clustered with the SAR11 strains in the maximum likelihood trees (Figure S1), but was placed distinct from the SAR11 group with the Bayesian method (Figure 1). 

**Table 1:** SAR11 genomes included in the analysis.

**Strain**	**Sampling site**	**Genome size (kb)**	**GC (%)**	**Subclade^1^**
HTCC1002	Coastal, temperate	1323	29.8	Ia
HTCC1062	Coastal, temperate	1309	29.7	Ia
HTCC7211	Open ocean, subtropic	1457	29.0	Ia
HIMB5	Coastal, tropic	1343	28.6	Ia
IMCC9063	Ocean, arctic	1284	31.7	IIIa
HIMB114	Coastal, tropic	1237	29.6	IIIa
HIMB59	Coastal, tropic	1410	32.3	V

^1^Subclade affiliations as suggested by [5].

**Figure 2:**
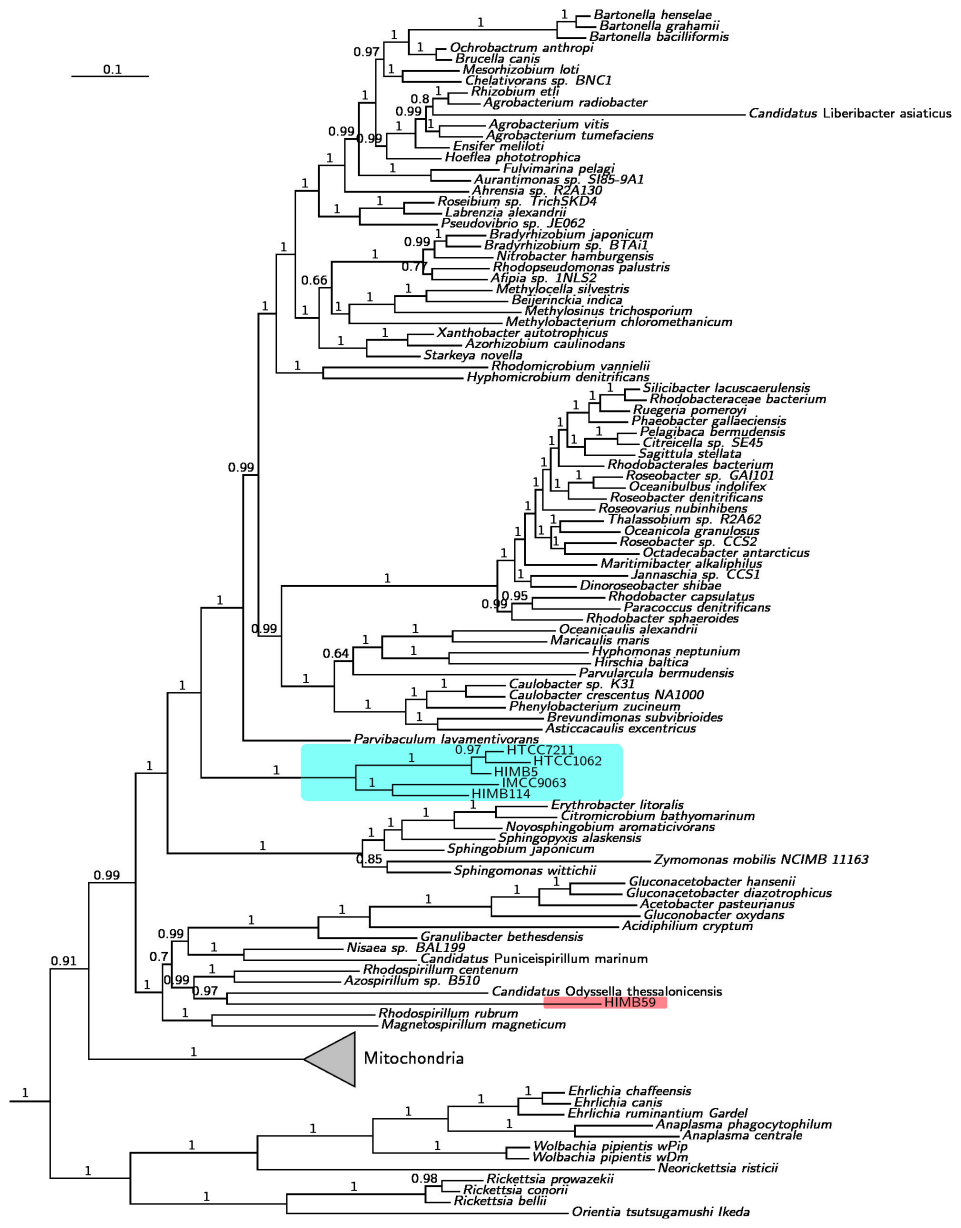
Phylogenetic analysis of the Alphaproteobacteria and mitochondria. Bayesian tree inferred with the CAT model applied to an alignment of 13 concatenated pan-orthologous proteins. HIMB59 (marked in red) is placed within the Rhodospirillales. The SAR11 clade (marked in blue) is placed within a broad group of free-living alphaproteobacterial species that includes the Sphingomonadales, Rhodobacterales, Rhizobiales, and Caulobacterales. Numbers at nodes show PP values.

 Next, we examined how the inclusion of mitochondrial sequences in the tree influenced the relation of HIMB59 to the SAR11 isolates. To this end, we made a separate clustering of proteins encoded by 48 mitochondrial genomes (Table S1). These were then merged with the corresponding clusters generated from the alphaproteobacterial genomes. First, we inferred the relationships of these taxa from a concatenated set of 29 proteins that indicated monophyly of the mitochondria in single protein trees with the maximum likelihood method. HIMB59 clustered separately from the SAR11 clade in these trees irrespectively of the method used for the analysis (data not shown). From this set, we selected a smaller set of proteins that individually supported the monophyly of mitochondria with more than 70% bootstrap support and included taxa such that each alphaproteobacterial genus would be represented by at least one species. This resulted in a dataset of 177 taxa and 13 orthologous protein clusters (Table S2). In the trees inferred from these proteins, both the Bayesian (Figure 2) and the maximum likelihood analyses (Figure S2) suggested that the SAR11 clade was placed distinct from the Rickettsiales and mitochondria and positioned within the free-living Alphaproteobacteria. Importantly, the SAR11 isolates of subtypes Ia and IIIa formed a clade with 100% support, while HIMB59 was positioned within the Rhodospirillales in the Bayesian analyses (posterior probability = 1.0) or at the base of the free-living Alphaproteobacteria in the maximum likelihood analysis (bootstrap support = 80%). Thus, none of the trees that included mitochondrial taxa suggested a placement of either mitochondria or HIMB59 within or as sister groups to the SAR11 clade. 

**Figure 3:**
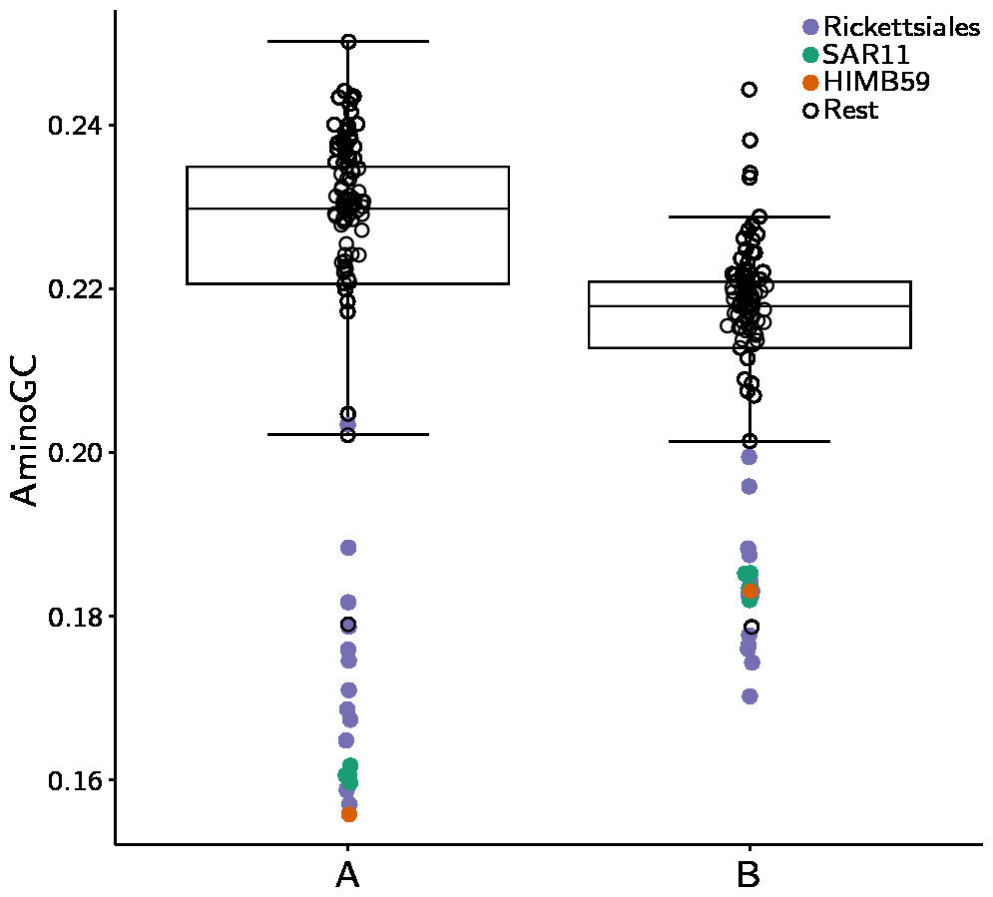
Influence of compositional bias. Box plots of aminoGC distribution among genes estimated from the concatenated alignments used to infer the phylogenies of the Alphaproteobacteria presented in Figures 1 and 2. A shows the distribution of the 150 proteins used in Figure 1 and B shows the 13 proteins used in Figure 2.

 There are extreme variations in G + C contents exist within the genomes of Alphaproteobacteria, ranging from 28% to 70%. In cases of strong heterogeneity in base composition patterns, non-phylogenetic signals in the data may override the true phylogenetic signal, resulting in different tree topologies for different data sets. To test the influence of AT/GC bias, we calculated the frequencies of amino acids coded by codons with GC in the first two positions (aminoGC) for both of the two concatenated data sets. The results showed that the concatenated alignment of the larger data set of 150 proteins was more strongly influenced by AT/GC bias than the smaller data set consisting of 13 proteins (Figure 3). The clustering of HIMB59 with the SAR11 genomes in the maximum likelihood analysis of the 150 proteins may thus represent such a nonphylogenetic signal, while the separation of HIMB59 from the SAR11 group of bacteria, as suggested from the smaller data set is more likely to represent the true phylogenetic signal. 

### Genome contents and architectures

#### HIMB59 and SAR11 differ in gene content

We used the clusters generated with orthoMCL to compare the gene content between HIMB59 and the other alphaproteobacterial genomes. A pairwise comparison was done between genomes of the SAR11 clade and the genomes of all other Alphaproteobacteria. As a similarity measure we used the number of shared orthologous clusters divided by the number of clusters in the smallest genome of the two (Table S3). As expected, all pairwise comparisons of members of subclades Ia and III in the SAR11 clade produced the highest similarity values (> 0.7) with each other. HIMB59 was a clear outlier, with a similarity value of 0.63, which was comparable to the values obtained in comparisons of the SAR11 strains with members of the Rhodobacterales and Rhizobiales. Vice versa, pairwise comparisons of HIMB59 with all other alphaproteobacterial genomes showed that *Nisaea* sp. BAL 199, a member of the Rhodospirillales (similarity value 0.77) and other members of the Rhodobacterales were most similar in gene content. In this comparison, HIMB5 and IMCC9063 were the most similar SAR11 genomes, but these were only the 63^rd^ most similar genomes overall to HIMB59. The least similar genomes to the SAR11 and HIMB59 genomes were members of the Rickettsiales (similarity values of about 0.4), consistent with the previously suggested independent reduction of genome sizes in SAR11 and the Rickettsiales [12]. We conclude that HIMB59 is less similar in gene content to members of the SAR11 clade than what it is to several members of the Rhodobacterales or Rhodospirillales. 

#### Gene synteny in HIMB59 and SAR11 is not conserved

**Figure 4:**
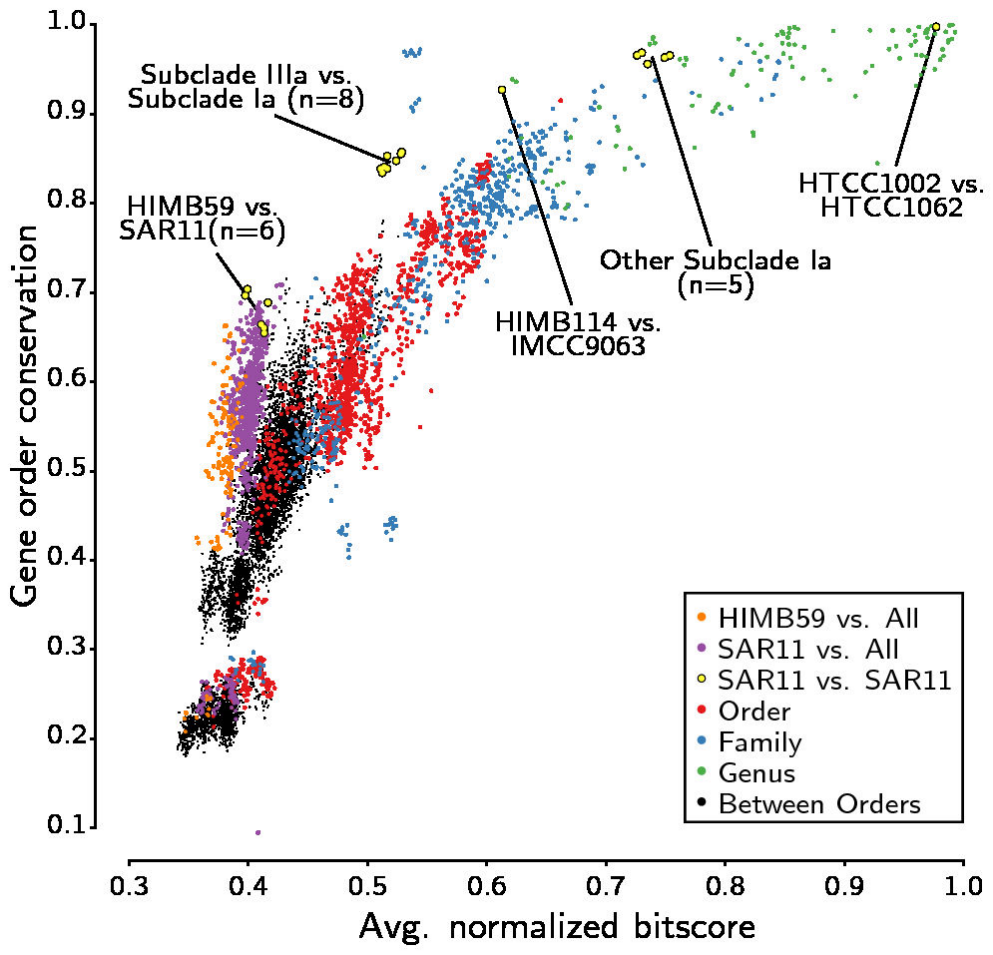
Gene order conservation plotted against protein sequence similarity for all possible pairs of the Alphaproteobacteria. The analyses of the SAR11 genomes highlighted as in [5]. The color-coding corresponds to the taxonomic assignments, with orange dots representing all pairwise comparisons of HIMB59 with all other taxa. Protein sequence similarity is defined as average normalized bit scores and gene order conservation as the fraction of syntenic genes.

Previous estimates of gene order comparisons indicated that the SAR11 strains (including HIMB59) showed much higher gene synteny related to genome sequence similarity than most other organisms [5]. But was the synteny between SAR11 and HIMB59 higher than between SAR11 and other members of the Alphaproteobacteria? To test this, we estimated pairwise synteny and identity values for all alphaproteobacteral taxa included in our phylogenetic analysis with the ruby synteny finder [14]. We classified all possible pairs of alphaproteobacterial taxa into four different sets: within genera, within families, within orders and between orders and the plotted identity and synteny level scores (Figure 4). Here, sequence identity was calculated as average normalized bit score of protein-coding genes, and synteny as the relative fraction of the number of genes with the same nearest neighbour divided by the number of shared genes. As expected, pairs of alphaproteobacterial taxa within the same genus showed much higher similarities than pairs within families and orders, although there were no clear boundaries. 

 The average amino acid identity of HIMB59 to subclade Ia of the SAR11 clade was estimated to 42%, ranging from 35% to 50% in the individual strain comparisons. SAR11-related pairs within subclade Ia and IIIa fell within the “within-genus” and “within-family” groups, respectively, while genome pairs between subgroup Ia and IIIa approached levels obtained for “within-order” comparisons. However, in all cases, the degree of gene order conservation for the SAR11 strains was among the highest for the particular sequence identity range. This suggests that members of the SAR11 clade have diverged more rapidly in sequence than in structure compared to other alphaproteobacterial genomes. Pairs of unrelated taxa had average bit scores less than 0.53 and synteny levels less than 0.78. Notably, all comparisons of sequence similarity of HIMB59 to the other SAR11 strains was within the range observed for pairs of unrelated Alphaproteobacteria, although synteny levels are in the upper range of unrelated taxa and comparable to those observed for bacterial pairs of the same order. Importantly, these values were similar to the synteny and identity values obtained in pairwise comparisons of *Ca*. Pelagibacter ubique to all other alphaproteobacterial taxa. Thus, HIMB59 genome is not more similar in gene order conservation to SAR11 than what it is to most other alphaproteobacterial genomes.

 Interestingly, we identified 2 outliers in the synteny-amino acid identity comparison plot. *Erythrobacter litoralis* and *Citromicrobium bathyomarinum* represents one such outlier. These two species are supposed to be related at the level of the order, but their high similarity (synteny = 0.92; bitscore = 0.66) suggests that they should at least be in the same family. Indeed, they form a monophyletic group in the phylogenies (Figures 1 and 2). The other outlier is *Rhodospirillum rubrum* and *Rhodospirillum centenum*, which are supposed to belong to the same genus. In this case, the low conservation of synteny and protein identity (synteny = 0.65; bitscore = 0.50) suggests that they should belong to different genera. Consistently, they do not form a group in our phylogenetic trees (Figures 1 and 2). Also their lifestyles are very different since *R. centenum* is a thermophile, isolated from a Yellowstone hot spring and has a genome with a G + C content of 70.5%.

#### Hypervariable region 2 (HVR-2) is missing in HIMB59

**Figure 5:**
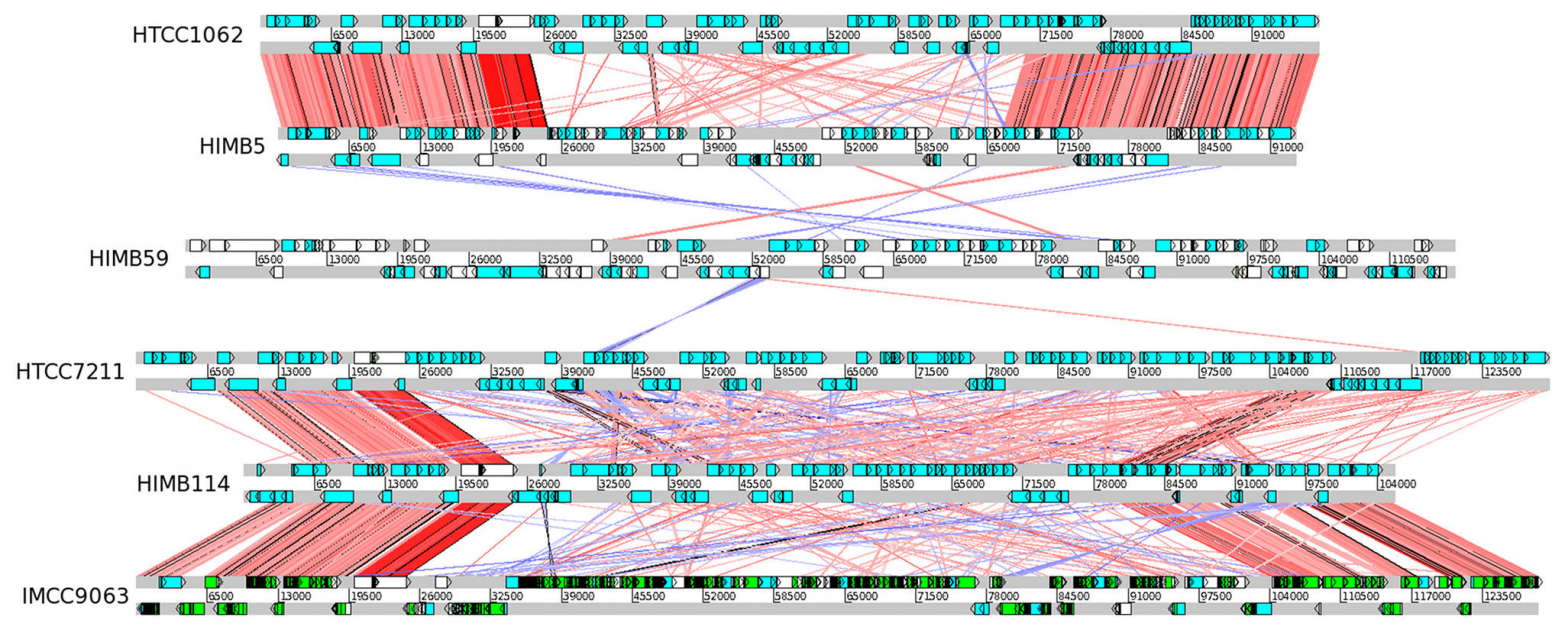
Visual representation of sequence similarity of HVR2. For all species except HIMB59 the region is located between 23S rRNA and 5S rRNA and includes 20kb on either side. For HIMB59, we extracted the region specified in [5] as the homologous region to HVR2 and included and an extra 20 kb on either side. The sequence similarity between any two regions is based on tblastx comparisons with an e-value threshold of E<10^−10^.

The genome of *Ca*. Pelagibacter ubique contains four highly variable regions (HVR 1-4). The 16S and 23S rRNA genes are located on one side of HVR2 and the 5S rRNA gene on the other [4]. Likewise, the ribosomal RNA genes were identified at approximately 100 kb downstream from the dnaA gene with an insertion separating the 16S-23S rRNA genes from the 5S rRNA gene in the other SAR11 genomes [5]. In contrast, in the HIMB59 genome, all rRNA genes are co-located and situated inside a single operon. In this genome, the HVR2-region was instead defined as a region flanked by a tRNASer and a tRNA^Ala^ genes. We compared the HVR2 region of the SAR11 genomes to the region flanked by the two tRNA genes in the HIMB59 genome by a tblastx-search and visualized the results with the Artemis Comparison Tool (Figure 5). We found no sequence similarity between genes in this region of the HIMB59 chromosome with genes located in HVR2 of the SAR11 genomes.

## Discussion

Our phylogenetic analyses suggest that HIMB59 is not a member of the Pelagibacterales. However, the placement of HIMB59 in relation to SAR11 in the phylogenies was sensitive to both the data sets and the methods used, as shown in table 2. With the more biased dataset of 150 proteins, all taxa with AT-rich sequences (Rickettsiales, SAR11 and HIMB59) were attracted to each other. In contrast, with a smaller dataset of 13 less biased proteins, the phylogenetic trees suggested a distinct placement of HIMB59 near to free-living Alphaproteobacteria. We have previously shown that the clustering of SAR11 was an artefact of strong AT mutation bias that could be resolved by the use of a carefully selected gene set [12]. Likewise, we suggest that the clustering of HIMB59 with SAR11 was an artefact of AT mutation bias, and that the observed clustering with the Rhodospirillales most likely represents its phylogenetic position. Based on these results, we suggest that SAR11 and HIMB59 have evolved small and AT-rich genomes independent of each other. 

**Table 2:** Placement of SAR11 and HIMB59 for different sets of orthologous clusters.

	**Position of SAR11**		**Position of HIMB59**	
**Cluster size**	**Bayesian**	**ML**	**Bayesian**	**ML**
13	RRC^a^ 1.0	RRC^a^ 84	Rhodo^c^ 1.0	Free^d^ 88
29	RRC^a^ 1.0	RRC^a^ 67	Free^d^ 0.9	Free^d^ 92
150	RRC^a^ 0.8	Rick^b^ 100	Rick^b^ 0.83	SAR11^e^ 100

^a^RRC = at the base of Rhizobiales, Rhodobacterales and Caulobacterales

^b^Rick = at the base of the Rickettsiales

^c^Rhodo = within Rhodobacterales

^d^Free = at the base of the free-living Alphaproteobacteria

^e^SAR11 = at the base of the SAR11 clade

 These results are fully consistent with a recent phylogenetic study [13], which showed convincingly that the clustering of HIMB59 within the SAR11 group was an artefact due to the shared, strong AT mutational bias in these genomes. To reduce the effects of the amino acid composition bias the authors recoded the dataset using the Dayhoff classes [15] and applied the CAT site heterogeneous mixture model to the recoded dataset. These measures dramatically changed the position of HIMB59 such that it was placed distantly from the SAR11 group of bacteria. 

 The separation of HIMB59 from the SAR11 clade, as suggested in our analysis as well as in the analysis of [13], is however in conflict with a recent study which claims that the conservation of gene content, synteny, and the HVR2 region provides a different source of evidence for a shared common ancestry of HIMB59 and the other SAR11 strains [5]. In this study, we have investigated whether the similarity of gene content and structure is higher than expected by chance through comparisons with other alphaproteobacterial genomes. Our results clearly showed that gene content is not more similar between HIMB59 and SAR11 than between SAR11 and other genomes of Rhodospirillales and Rhodobacterales. Indeed, HIMB59 differs in several metabolic pathways that are considered to be characteristic of the SAR11 clade as pointed out by [5]. For example, HIMB59 lacks genes for the glyoxylate bypass present in the other genomes, but has a complete Embden-Meyerhof-Parnas glycolysis pathway unlike the SAR11 genomes. Moreover, our study has shown that the HIMB59 genome has an equal level of gene order conservation with genomes of SAR11 genomes as it does with several genomes within the Rhodobacterales. Finally, the rRNA genes used to define HVR2 in the SAR11 clade are located elsewhere in the HIMB59 genome, and there is no sequence similarity between HIMB59 and the other SAR11 genomes in the region positioned at approximately the same distance from dnaA as HVR2 in the SAR11 genomes. Thus, all genomic data accumulated to date, whether it is based on phylogenies, gene content, gene order or hypervariable regions, suggest that HIMB59 is not monophyletic with the other strains in the SAR11 clade.

 Additionally, our phylogenetic analysis suggests that neither the SAR11 clade nor HIMB59 is specifically related to the Rickettsiales. Despite very similar genome sizes, gene content measures consistently indicated that the marine and intracellular groups of bacteria with small genome sizes have undergone genome reduction independently of each other, resulting in strikingly different gene pools. In fact, we have shown here that both HIMB59 and members of the SAR11 clade are more similar in gene content to free-living soil and marine bacteria with large genomes than to the Rickettsiales. Finally, our phylogenetic analysis has conclusively shown that mitochondria is neither related to the SAR11 group of bacteria nor to HIMB59 since none of our protein trees suggest such a relationship. In addition to the phylogenies presented here, two previously published papers [11,13] have come to the exact same conclusion. 

 At first sight, these results are puzzling since three other phylogenetic studies concluded that mitochondria share a common ancestor with the SAR11 clade [8–10]. Mitochondria, Rickettsiales and the SAR11 group of bacteria are similar in that they all have genomic A + T content values higher than 60%, as compared to less than 40% for most other alphaproteobacterial genomes (see Figure 1 in [13]). A grouping of AT-rich taxa is a well-known artefact of strong compositional biases in the data, and it has been argued that the occasional clustering of SAR11 with mitochondria and/or Rickettsiales represents precisely such an artefact [11–13]. Hence, if the problems posed by these biases are not addressed through careful selection of genes and models in the phylogenetic analysis, the effects of the compositional biases can easily override the underlying phylogenetic signal.

 In the phylogenetic analysis by Thrash et al. ([9]), no attempts were made to choose genes less affected by these compositional biases, or to test for the presence of such biases in the datasets. The phylogenomic analyses of the Alphaproteobacteria were based on relatively small datasets with up to 60% of the taxa missing. Moreover, the clustering of orthologs yielded only 15 orthologous clusters in a dataset of 127 alphaproteobacterial genomes although 10% of missing taxa was allowed. In contrast, our clustering process identified 75 pan-orthologous clusters in 135 alphaproteobacterial genomes, including only single copy genes and no missing data. None of the pan-orthologs identified in our study were present in the 15 clusters used by Thrash and collegues ([9]) as the most taxon-inclusive dataset, which questions the validity of the HAL pipeline for building protein clusters. This pipeline was initially developed for comparative analysis of yeast and closely related bacterial genomes [16], and we speculate that the settings used may not have been appropriate for the identification of orthologous groups across more distantly related genomes.

 Moreover, an inspection of the proteins in the mitochondrial clusters used in ([9]) revealed that these were mostly mitochondrial ribosomal proteins, which tend to evolve rapidly and as we have shown previously, are especially sensitive to AT/GC mutation biases [11]. In particular, the few proteins that are robust to these biases, such as Cytochrome oxidase subunit 1 and Cytochrome *b* [11], were not included in the analysis. Thus, the lack of manual curation of the automatically generated mitochondrial and alphaproteobacterial protein clusters may have led to the inadvertent inclusion of proteins for which the noise derived from the compositional bias was stronger than the signal derived from their evolutionary relationships.

 Another striking difference is that the phylogeny reported by Thrash and collegues ([9]) clusters HIMB59 with the SAR11 group. As discussed above and in Rodriguez-Ezpleta and Embley ([13]), such a clustering is most likely also a long-branch attraction (LBA) artifact. The HIMB59 genome is not only very AT-rich, but it has also evolved very rapidly in sequence, resulting in a relatively low ratio of sequence similarity to gene synteny compared to other bacteria. Consistently, in our phylogenetic analysis, HIMB59 is situated on a long branch. However, in the tree presented in Figure 6 in [9], HIMB59 is situated on a surprisingly short branch. Upon further inspection, it seems as if the branch lengths that exceed a value of 1 have lost their integer part. Thus, we believe that in the case of HIMB59 the real branch length should be 1.22, but has been inadvertently changed to 0.22. Finally, Thrash and collegues claim that they have used the CAT model of rate heterogeneity in RAxML [17] to deal with LBA and provide a reference to Lartillot et al. [18], which shows how the CAT model is able to accommodate such biases. However, the Lartillot paper refers to the CAT model implemented in Phylobayes, which is a site-heterogeneous model that models different stationary frequencies across sites [18], whereas the CAT model that is implemented in RAxML is an approximation of the gamma distribution for rate heterogeneity used to speed up the tree-search algorithm.

 The identification of divergence patterns for deeply diverging clades is notoriously difficult and prone to artefacts. The main problem is that the rate of sequence evolution is often too high to accurately trace evolutionary events over long distances. Differences in the patterns of substitutions among lineages represent another well-known source of artefacts in phylogenetic inferences. The difficulties with sequence-based approaches to infer evolutionary relationships have enforced the use of alternative sources of data such as gene content, gene synteny and character sites as markers of shared history. Regarding HIMB59, all sources of data suggest that its phylogenetic position is distinct from the SAR11 group of bacteria.

 What then is HIMB59 related to? The study by Rodrigues and Embley [13] suggests that HIMB59 clusters with *Candidatus* Puniceispirillum *marinum* IMCC1322, which is the first cultivated member of the SAR116 clade affiliated with the Rhodospirillales. Although, our phylogenies do not identify the closest relative of HIMB59 with certainty, it is notable that both the Bayesian and the maximum likelihood phylogenetic analysis of the smallest dataset indicate a clustering with *Odysella* within the broader group of the Rhodospirillales that also includes the SAR116 clade (Figures 1, 2). *Odysella* was not present in the paper by Rodriguez and Embley [13], and such a clustering might therefore have been missed in their analysis. The tentative placement of HIMB59 with SAR116 and the Rhodospirillales is intriguing since the SAR116 clade is abundant in various marine environments based on culture-independent approaches. Single cell genomic technologies offer great promises and may generate data to help elucidate the phylogenetic relationships of the currently available bacterial isolates with the many bacteria that have as yet not been cultivated from marine ecosystems.

## Methods

### Datasets

A set of 135 representative Alphaproteobacterial species was selected from the JGI ftp site as of 2011-08-04 (release img_w_v340). We included at least one alphaproteobacterial species for each genus, with priority given to complete genomes (see Table S1). IMCC9063 was downloaded from the NCBI ftp site at 2011-08-04, HIMB5 from the website http://moore.jcvi.org/more / at 2011-08-04, HIMB59 from the Camera website at 2011-08-10 and *Ca*. Odysella *thessalonicensis* from the NCBI ftp site at 2011-10-05. Included in the analyses were all mitochondrial taxa in Trash et al. [16] plus a few additional taxa to get a better coverage of the mitochondrial grouping as detailed in Table S1. 

### Orthologous clusters

The protein-coding genes of the Alphaproteobacteria were clustered with orthoMCL using default parameters. We included for analysis all orthologous groups that contained at least 130 (out of 135 species), no more than 3 copies per genome and no more than 142 genes per cluster. Of these clusters, 74 contained pan-orthologs, with exactly one copy per genome. We added a few orthologous groups included in Viklund et al. [12] and Williams et al. [8] and divided a cluster containing rpoB and *rpoC* into two separate clusters following an alignment with mafft (version 6.864b – [19]) and a tree with RAxML (version 7.2.8 - [20]). A hidden markov model (HMM) was built with hmmer based on an alignment of the ingroup sequences. The HMM profile was then used to search for related proteins among the outgroup taxa (E-value < 0). At least one copy per outgroup taxa were included as detailed in Table S1.

 The selected clusters were subjected to careful quality control. All clusters were aligned with mafft using the localpair option and the alignments were visually inspected. Missing sequences were added after a tblastx-search [21] against the genome of the missing species. We identified and merged genes that were frame-shifted. Mis-annotated start-codons were moved either upstream or downstream. RAxML trees were built for each cluster using the LG model [22] and 100 quick bootstraps. Whenever multiple copies from the same taxon (paralogs) were detected, both the tree and the alignment were inspected. Whenever these paralogs clustered together in the tree, the sequence with the shortest branch was retained. Copies that were placed within the outgroup were removed. Whenever two or more paralogs were placed at different positions in the alpha-proteobacterial tree, the whole cluster was removed. If two or more copies of genes from the same outgroup species were placed at different positions, we first inspected the alignment to see if any clear choice could be made, otherwise all copies were removed. Clusters in which the outgroup and ingroup were mixed were removed. This procedure left us with 209 clusters. After discordance filtering to remove potentially horizontally transferred genes [8], 150 orthologous groups were retained.

 All of the mitochondria were clustered using orthoMCL (version 2.0, [23]) in the same way as above (mitoCOGs). We then used mitochondrial genome of R. americana as our base for selecting mitochondrial genes. First, all proteins encoded by the *R. americana* mitochondrial genomes were used as queries in BLAST searches against the alphaCOGs. Whenever multiple hits of equal strength were obtained to several alphaCOGs, the proteins in these COGs were aligned with the *R. americana* protein sequence and used to construct a tree, and the alphaCOG that clustered with the *R. americana* protein in the tree was selected. Next, the genes from the mitoCOGs containing the corresponding *R. americana* gene were added to the corresponding alphaCOG. Outgroup and quality control was performed in the same way as described above with the addition that we selected preferentially mitochondria-encoded genes in those cases where both nuclear and mitochondrial encoded copies were detected. Only those clusters where the mitochondria formed a monophyletic clade were retained (29 OGs in total).

### Phylogeny

All aligments were filtered with Gblocks (version 0.91b, [24]) using default parameters and the most optimal model of evolution was estimated for each protein using ProtTest (version 3.2, [25]). The 150 single protein alignments were concatenated. RAxML was run on the alignment with a partitioned model (using the optimal model for each protein) with 19 independent searches for the best tree and 100 bootstrap replicates. Phylobayes (3.2f, [26]) was run with 6 chains for 12000 generations using the CAT model. The alignments including mitochondrial proteins were treated with Gblocks, but allowing for gaps in half of the sites. These were run using RAxML with 100 bootstrap replicates and 4 chains of Phylobayes for 68000 generations.

### Genome comparisons

Gene content similarity was estimated using the alphaproteobacterial orthologous clusters using the number of shared orthologous groups between two taxa divided by the number of orthologous groups in the taxa with the smallest genome. Synteny and amino acid identity for all alphaproteobacterial species were calculated with the ruby-scripts from Yelton et al. HVR2 and a region of 20 kb up and downstream of HVR2 were extracted from the SAR11 genomes. The region flanked by tRNA^Ser-GGA^ and tRNA^Ala-GGC^ was extracted from the HIMB59 genome. These regions were blasted against each other using tblastx and compared using artemis comparison tool [27].

## Supporting Information

Figure S1 **Phylogenetic analysis of the Alphaproteobacteria.** Maximum likelihood tree inferred from an alignment of 150 concatenated pan-orthologous proteins. HIMB59 clusters with the SAR11 clade. Numbers at nodes show bootstrap support values.
(PDF)

Figure S2 **Phylogenetic analysis of the Alphaproteobacteria and mitochondria.** Maximum likelihood tree inferred from an alignment of 13 concatenated pan-orthologous proteins. HIMB59 is placed at the base of the Alphaproteobacteria. Numbers at nodes show bootstrap support values.
(PDF)

Table S1 **List of taxa included in the phylogenetic analyses of Alphaproteobacteria and mitochondria.**
(XLS)

Table S2 **List of mitochondrial genes included in the phylogenetic analyses.** Ntaxa refers to the number of taxa included in the single protein trees. Support refers to the bootstrap support for monophyly of mitochondria in the single protein trees with the maximum likelihood method. A star next to the bootstrap support value indicates that the corresponding single protein tree had a bootstrap value higher than 70% in support for the monophyly of mitochondria.
(PDF)

Table S3 **Measures of gene content similarity in pairwise comparisons of alphaproteobacterial genomes.** The values for each pair of genomes corresponds to the number of shared orthologous clusters divided by the number of clusters in the smallest genome of the two.
(XLS)
