# Supplementary figures and images for: Comparative and Phylogenomic Evidence That the Alphaproteobacterium HIMB59 Is Not a Member of the Oceanic SAR11 Clade
Source: PLoS One. 2013 Nov 1;8(11):e78858. doi: 10.1371/journal.pone.0078858 (PMC3815206; doi:10.1371/journal.pone.0078858)

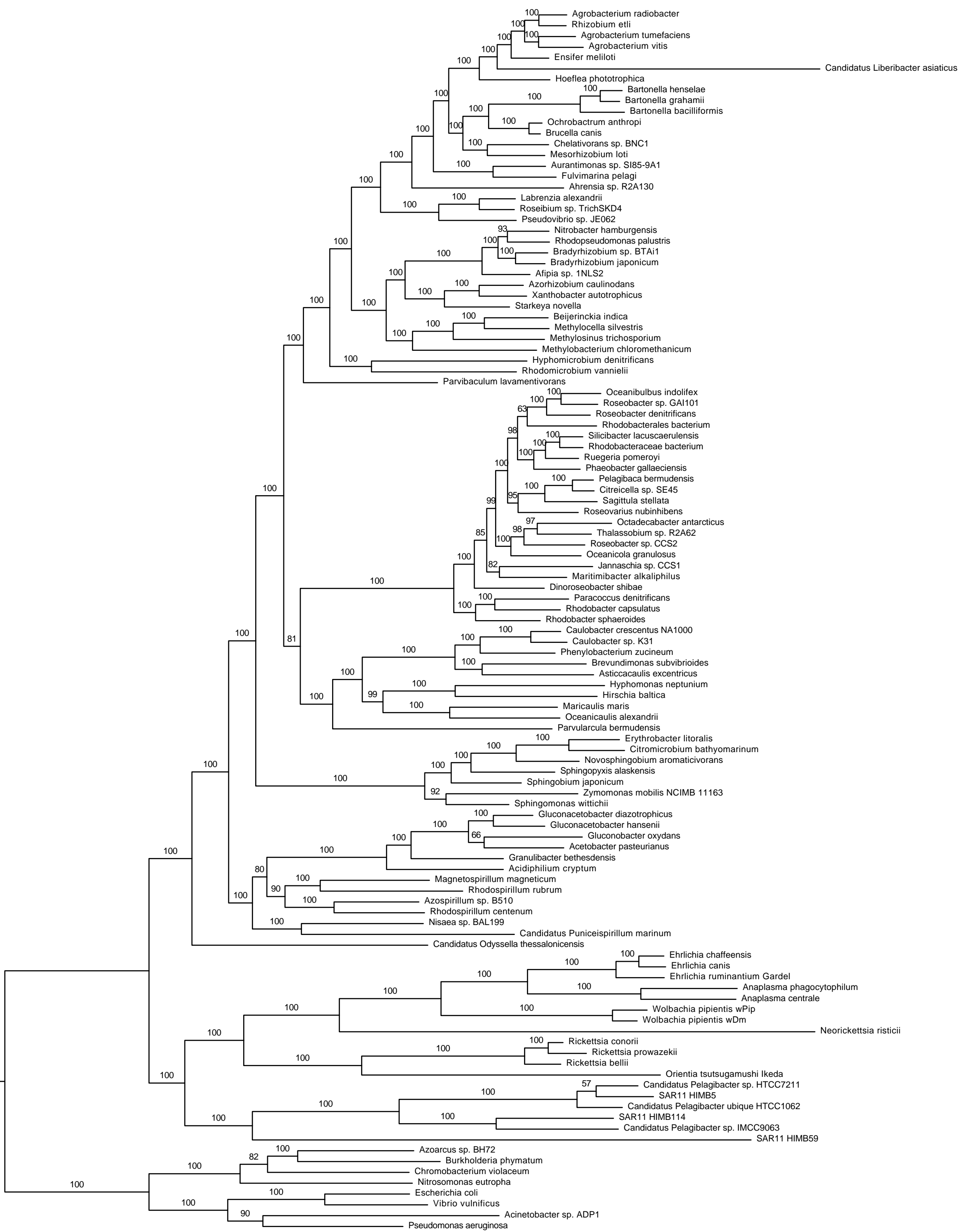

0.2

Supplement: Figure S1 — Phylogenetic analysis of the Alphaproteobacteria. Maximum likelihood tree inferred from an alignment of 150 concatenated pan-orthologous proteins. HIMB59 clusters with the SAR11 clade. Numbers at nodes show bootstrap support values. (PDF) [file pone.0078858.s001.pdf]

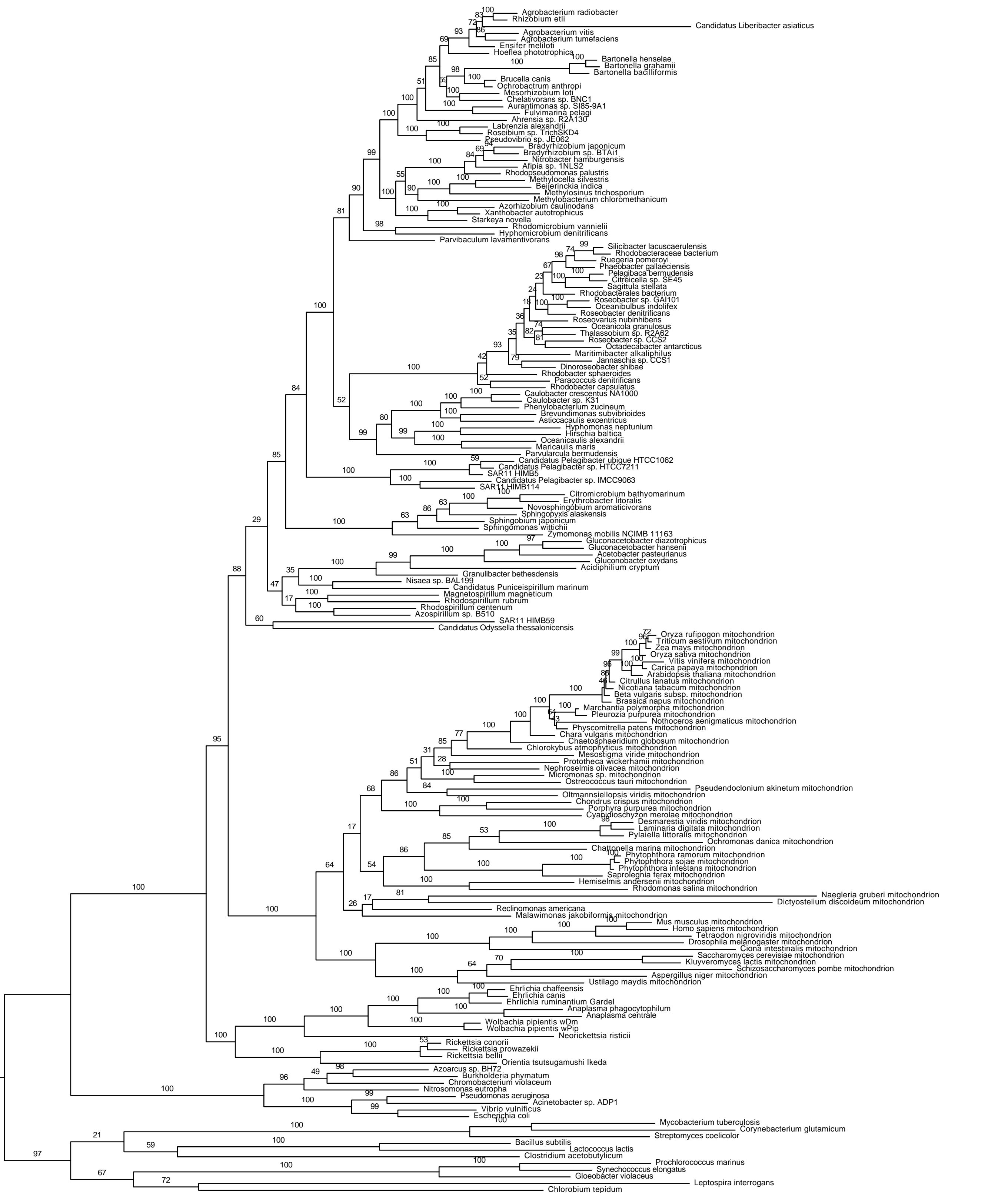

0.2

Supplement: Figure S2 — Phylogenetic analysis of the Alphaproteobacteria and mitochondria. Maximum likelihood tree inferred from an alignment of 13 concatenated pan-orthologous proteins. HIMB59 is placed at the base of the Alphaproteobacteria. Numbers at nodes show bootstrap support values. (PDF) [file pone.0078858.s002.pdf]
