# Supplementary material for: Comparative and Phylogenomic Evidence That the Alphaproteobacterium HIMB59 Is Not a Member of the Oceanic SAR11 Clade
Source: PLoS One. 2013 Nov 1;8(11):e78858. doi: 10.1371/journal.pone.0078858 (PMC3815206; doi:10.1371/journal.pone.0078858)
Supplement: Table S2 — List of mitochondrial genes included in the phylogenetic analyses. Ntaxa refers to the number of taxa included in the single protein trees. Support refers to the bootstrap support for monophyly of mitochondria in the single protein trees with the maximum likelihood method. A star next to the bootstrap support value indicates that the corresponding single protein tree had a bootstrap value higher than 70% in support for the monophyly of mitochondria. (PDF) [file pone.0078858.s004.pdf]

Gene	Ntaxa	Length	Support	
atp1	162	454	98	*
atp3	137	157	93	*
atp6	174	101	74	*
cob	167	266	97	*
cox1	159	440	96	*
cox2	158	130	95	*
cox3	157	171	70	
nad10	121	146	12	
nad11	125	345	83	*
nad1	163	254	54	
nad2	164	197	12	
nad4	163	310	92	*
nad6	157	107	81	*
nad7	150	350	88	*
nad8	118	148	18	
nad9	157	111	49	
rpl11	139	73	65	
rpl14	158	61	9	
rpl5	165	90	82	*
rpl6	155	88	7	
rps12	177	88	5	
rps4	138	116	16	
sdh2	125	181	73	*
tatC	147	131	100	*
